# Allocentric directional processing in the rodent and human retrosplenial cortex

**DOI:** 10.3389/fnhum.2014.00135

**Published:** 2014-03-17

**Authors:** Rebecca Knight, Robin Hayman

**Affiliations:** ^1^Department of Psychology, University of HertfordshireHatfield, UK; ^2^Department of Clinical and Experimental Epilepsy, Institute of Neurology, University College LondonLondon, UK

**Keywords:** head direction, retrosplenial cortex, extra-hippocampal, allocentric, interspecies

## Abstract

Head direction (HD) cells in the rodent brain have been investigated for a number of years, providing us with a detailed understanding of how the rodent brain codes for allocentric direction. Allocentric direction refers to the orientation of the external environment, independent of one’s current (egocentric) orientation. The presence of neural activity related to allocentric directional coding in humans has also been noted but only recently directly tested. Given the current status of both fields, it seems beneficial to draw parallels between this rodent and human research. We therefore discuss how findings from the human retrosplenial cortex (RSC), including its “translational function” (converting egocentric to allocentric information) and ability to code for permanent objects, compare to findings from the rodent RSC. We conclude by suggesting critical future experiments that derive from a cross-species approach to understanding the function of the human RSC.

Only a handful of behavioral studies have examined the role of head orientation in human navigation (Prévost et al., 2003; Wiener et al., 2011), yet a great deal is known about the function of head orientation in rodent navigation. The reason for this wealth of knowledge can be attributed to the discovery of head direction (HD) cells in the rodent brain in the mid 1980s (Ranck, 1984). Crucially, evidence is now emerging which suggests that similar allocentric mechanisms are also present in the human retrosplenial cortex (RSC; Baumann and Mattingley, 2010; Vass and Epstein, 2013). This review begins by providing a brief overview of the HD literature, followed by a discussion of the recent human findings that implicate the RSC and dorsal presubiculum. After a more detailed examination of the RSC, we conclude that in both rats and humans, the RSC is involved in integrating self-motion cues with stable, distal landmark cues so that egocentric viewpoints can be mapped onto an allocentric frame of reference. The review ends with a discussion of how these inter-species similarities can be applied to future questions about allocentric directional processing.

## Neural correlates of allocentric directional processing

Taube et al. (1990a,b) published the first quantitative measurements of HD cells in the rat postsubiculum (PoS—sometimes referred to as the dorsal presubiculum). Along with place cells coding for location (O’Keefe and Dostrovsky, 1971) and grid cells coding for distance (Hafting et al., 2005), HD cells provide the neural correlate for an allocentric, cognitive map (O’Keefe and Nadel, 1978). In particular, HD cells provide allocentric directional information that is crucial for forming a map rather than a route-based representation. In this early work, Taube et al. noted that each HD cell is specific to a single head (but not body) orientation and when the head is directed to the cell’s preferred firing direction, the cell fires at maximum. Collectively these cells are thought to encode for all possible heading directions.

As a result of further investigation, it is now thought that the HD pathway involves multiple brain structures broadly comprising the Papez circuit (Taube and Bassett, 2003). These areas include the subiculum (Taube et al., 1990a,b), the anterior thalamic nuclei (ADN; Blair and Sharp, 1995; Taube, 1995; Taube and Muller, 1998), the dorsal tegmental nuclei (DTN; Bassett and Taube, 2001), the lateral mammillary nuclei (LMN; Blair et al., 1998; Stackman and Taube, 1998) and the RSC (Chen et al., 1994a; Cho and Sharp, 2001). Research has also focused on the type of information utilized by the HD system, namely landmark and self-motion cues. Studies examining the influence of landmarks on HD firing have shown that external cues can exert strong control over HD firing (Chen et al., 1994b; Taube, 1995; Stackman and Taube, 1997), but that this control is variable depending upon the type of cue (Taube et al., 1990b; Knight et al., 2011; Clark et al., 2012), the location of that cue in the environment (Zugaro et al., 2001), the prior experience and the duration of exposure to that cue (Goodridge et al., 1998). HD cells are also thought to rely on self-motion cues (Taube and Burton, 1995; Stackman and Taube, 1997; Stackman et al., 2003; Yoder et al., 2011). For example, HD cells will continue to fire in the dark (albeit with a drift in preferred firing direction, Taube et al., 1990b).

Although the majority of this work has focused on rodent recordings, HD cells have crucially been recorded in the presubiculum of freely moving primates (Robertson et al., 1999). The clear existence of HD cells in primates indicate that the existence of HD cells in humans is likely, but to date the closest evidence we have comes from two separate fMRI studies (Baumann and Mattingley, 2010; Vass and Epstein, 2013).

Baumann and Mattingley (2010), who allowed participants to view pairs of landmarks within a newly learnt virtual reality maze, conducted the first of these studies. These landmark pairs were either matched for allocentric direction (e.g., north–north) or not (e.g., west–north). They exploited the principle that repeated exposure to a stimulus will lead to attenuation in the fMRI (BOLD) response. If another stimulus is presented and is deemed sufficiently different from the first stimulus, the BOLD response should increase. Using this neural adaptation method, they found that activity within the medial parietal cortex (specifically, Brodmann area 31) was modulated by changes in allocentric heading direction. In addition, Vass and Epstein (2013) scanned students whilst they viewed photographs of highly familiar campus locations. Using multivoxel pattern analysis (MVPA) they compared the pattern similarity of the same (e.g., north–north) and different (e.g., west–north) allocentric directions and found that the presubiculum was able to differentiate between directions. They also used a neural adaptation method and interestingly found the opposite to Baumann and Mattingley, in that there was anti-adaptation in the retrosplenial complex. As the authors suggest, the anti-adaptation does not necessarily mean an absence of HD signal in RSC. Given that photographs were used (with a number of landmarks, none of which were “distal” in the sense of being projected at infinity) the HD cells may have used a variety of landmarks on different occasions, creating an anti-adaptation effect. Also, participants in this study were highly familiar (3 years exposure) with the environment compared to the 5 h training in the Baumann and Mattingley study. It is thought that the RSC has a greater response to highly familiar locations (Epstein et al., 2007), thus the RSC activation in response to familiarity may have masked any adaptation effects.

Taken together, these studies implicate both the medial parietal lobe (Baumann and Mattingley, 2010) and the medial temporal (Vass and Epstein, 2013) lobe in computing allocentric direction. Given what we know about the HD circuitry in rats, it is perhaps not surprising that (even from just two studies) different areas of the human brain have been associated with allocentric direction. However, both fMRI studies failed to report many brain areas that are implicated in the rodent HD system. One possible explanation for this is that directional information in the fMRI studies was only provided by visual as opposed to self-motion cues. Many areas in the HD circuit such as the LMN and DTN are thought to receive direct input from the motor cortex (Brown et al., 2005; Biazoli et al., 2006). A lack of head movement within the scanner would perhaps prevent any directional information from reaching these equivalent areas in the human brain.

In contrast, HD cells in the rodent RSC and PoS appear to be particularly sensitive to visual information. Indeed, Goodridge and Taube (1997) found that when the PoS was lesioned, HD cell firing continued in the ADN but the control visual landmarks had over the ADN HD cells was disrupted. This is also true when the RSC is lesioned and recordings are taken from the ADN (Clark et al., 2010). Thus, the findings from both Baumann and Mattingley and Vass and Epstein seem to concur with the recordings of HD cells in the rat RSC (Cho and Sharp, 2001) and PoS (Taube et al., 1990a,b), respectively.

## Role of the retrosplenial cortex

Before we make a focused interspecies comparison of the RSC, it is important to consider the potential issues of comparing a BOLD signal with the spiking rate of single cells. Correlations between the two measures are known to be variable, particularly in deep structures such as the hippocampus (Ekstrom et al., 2009; Ekstrom, 2010). This review will not attempt to provide a direct discussion of this topic, but nevertheless it is important to employ caution when making methodological comparisons such as the ones in this review (for a more detailed treatment see Logothetis, 2008).

Another important issue to consider before making any interspecies comparisons is the distinction between the human RSC (Brodmann areas 29 and 30) and the retrosplenial complex, a larger area that also encompasses the remaining posterior cingulate cortex (Brodmann 23 and 31) (Vann et al., 2009). Defining the RSC and distinguishing it from the other structures in the posterior cingulate cortex is often regarded as a complex issue (Vann et al., 2009). Indeed, Auger et al. (2012) noted that some fMRI studies found activation not in the area that was claimed to be the RSC, but rather an area located more posteriorly and superiorly in the posterior cingulate cortex.

Despite the complexities of recording from the human RSC, one finding that has a recurring place in the literature is that the RSC is responsible for processing spatial information during navigation (Aguirre et al., 1996; Spiers et al., 2001; Rosenbaum et al., 2004; Ino et al., 2007; Vann et al., 2009). Most recently, Auger et al. (2012) found that the RSC was more active for landmarks that were permanent, irrespective of object size. Using fMRI, they scanned participants whilst they viewed images of single landmarks. They found that parahippocampus responded to a large proportion of different landmarks but the RSC was active for only the most permanent landmarks (e.g., telephone box, lighthouse, etc.) rather than less permanent landmarks (e.g., bike, bus etc.). This finding is interesting on a number of levels. As mentioned above, HD cells recoded in the rat RSC (Chen et al., 1994a; Cho and Sharp, 2001) are thought to be particularly sensitive to visual information, namely landmarks (Goodridge and Taube, 1997). Moreover, Zugaro et al. (2001) found that background cues are more likely to influence HD firing compared to foreground cues. In a natural environment, distal objects on the horizon (trees, buildings, the sun) tend to be more permanent than near objects. It thus appears that the RSC in humans and HD cells in rats are particularly sensitive to permanent objects. It is important to note that these HD cells were recorded in the ADN, nevertheless it appears that the characteristics of HD cells found in rats is congruent with findings from the RSC in humans. Indeed lesions to the RSC in rats disrupts the learning of distal visual cues (Vann and Aggleton, 2005).

Auger et al.’s (2012) findings also appear consistent with the prominent theory that the RSC has a translational function. This translational theory suggests that the RSC is responsible for translating egocentric information to allocentric information (Byrne et al., 2007; Epstein et al., 2007; Lambrey et al., 2012; Zhang et al., 2012) and some suggest vice versa (Iaria et al., 2007). In a virtual reality fMRI task, Lambrey et al. (2012) found that the RSC was active when participants had to imagine rotating their viewpoint around an array of four objects on a table. They therefore concluded that the RSC played a role in taking an egocentric viewpoint and updating it in an allocentric reference frame, a finding congruent with Vass and Epstein (2013).

This translational theory of the human RSC demonstrates some interesting parallels with the rodent RSC if we start to examine the transmission of information in the rat HD circuit. Studies have shown that the rat RSC receives direct visual and self-motion inputs. With regards to visual inputs, the RSC has direct connections to visual areas (Vogt and Miller, 1983). The self-motion signal is thought to reach the RSC via strong reciprocal connections to the PoS (Wyss and Van Groen, 1992), which in turn is highly connected to the ADN. The HD signal sent from the ADN is thought to contain vestibular and proprioceptive information, that is initially processed in the LMN (Blair et al., 1998) and DTN (Bassett and Taube, 2001). In fact, RSC HD cells lose some directional sensitivity when there is a lack of head movement (Chen et al., 1994b). The rat RSC therefore appears to integrate visual and self-motion cues in order to produce a directional signal. The visual component of this signal is likely to contain allocentric information as the formation of a cognitive map or an allocentric viewpoint of space is thought to depend on the processing of visual information, particularly an array of distal landmarks (O’Keefe and Conway, 1978; Morris, 1981; Muller and Kubie, 1987; Wiener et al., 2002). Self-motion information can then be used to update a viewpoint-dependent egocentric location within an environment (Burgess, 2006; Taube, 2007). If RSC HD cells are integrating visual and self-motion cues, then the idea that the human RSC is responsible for translating egocentric information to an allocentric frame appears compatible.

By examining the data from both species, it appears that the RSC is involved in integrating self-motion cues with stable, distal landmark cues so that egocentric viewpoints can be mapped onto an allocentric frame of reference. We now turn our attention to looking at how this apparent overlap can be applied to future questions about allocentric directional processing.

## Future directions

Although there have been relatively few single cell recordings of the rat RSC during navigation tasks, there are some key findings that have yet to be fully explored in the human field. Cho and Sharp (2001) recorded 120 cells in the RSC of 25 rats. They replicated previous recording of HD cells in this area (Chen et al., 1994a,b), but interestingly they also recorded direction dependent place cells and cells that showed a correlation with running speed. These cells appear to further strengthen the link between the function of the rat RSC and the translational theory. Within the rat RSC, there are cells that can code directly for egocentric movements (running speed) and allocentric space (HD cells and direction dependent place cells). A combination of these cells makes the RSC an ideal structure to translate egocentric information onto an allocentric frame of reference. Based on this rodent paper, it would be interesting to see if the human RSC is also able to code for running speed. There is evidence to suggest that the human RSC is sensitive to the speed of egocentric movements. In a study designed to look for evidence of grid cells in humans (Doeller et al., 2011), the RSC showed significant adaptation to running direction. Interestingly, they found that this adaptation was greater for fast rather than slow runs (median split). In order to investigate this further, an fMRI study could be designed to test specifically whether activity in the RSC shows a linear correlation with a gradual increase or decrease in movement velocity. It would however be necessary to control for an increase in arousal that may occur as a result of increasing the flow of the visual field. A control condition could be implemented where the movement of the field was random but the rate of flow matched the coherent movement in the experimental manipulation.

Cho and Sharp (2001) also found cells in the rat RSC that were sensitive to the angular movement of the rat’s head. These angular head velocity cells have also been found in the ADN (Blair and Sharp, 1995), the LMN (Stackman and Taube, 1998) and the DTN (Bassett and Taube, 2001). Essentially the HD cell’s peak firing direction will shift anti-clockwise when the rat moves their head in a clockwise movement, but shift clockwise during anti-clockwise movements. This anticipatory firing implies that HD cells in RSC (and other regions) code for the future position of the rat’s head. Currently, it is unclear if the human RSC plays a role in anticipatory head movement, although we do know that the head movement itself has an anticipatory function during navigation (Prévost et al., 2003). The task of finding the neural correlate of angular head velocity in humans would certainly have its challenges. Using fMRI would not be ideal given the poor temporal resolution and the inherent restriction of head movement. The latter issue could be overcome by providing the necessary optic flow that would indicate a head movement in space. The former issue could be overcome by exploiting not only the sensitivity of RSC HD cells to direction but also the idea that they are modulated by the movement of the rat’s head on the approach to that direction. We know that fMRI activity in the human RSC shows adaptation when people are shown still images of landmarks matched for allocentric direction (e.g., north–north) rather than mismatched (e.g., north–west) (Baumann et al., 2012; Vass and Epstein, 2013). An fMRI study could therefore be designed where the target landmark comes into view by rotating the visual scene. If the rotations were matched across a pair of trials (e.g., they are currently facing east and move in a counterclockwise rotation to face the north landmark) then adaptation may occur in the RSC. If however, there were a mismatch in rotation (one movement is counterclockwise from east and the other is clockwise from west) then perhaps adaptation in the RSC would not occur. In addition, MVPA could assess whether some brain regions are better at predicting fast versus slow rotations and if this is moderated by the direction of rotation (clockwise versus counterclockwise).

## Concluding remarks

As is true of many research fields, studies targeting a species are often designed in light of prior research on that specific species. This approach is often very sensible given the anatomical difference between species and the restriction of species-specific methodological tools. However, we hope that by giving particular focus to the RSC we have demonstrated that in both rats and humans, this brain area is involved in integrating self-motion cues with stable, distal landmark cues so that egocentric viewpoints can be mapped onto an allocentric frame of reference. In our opinion, this agreement should be used as a platform to formulate new research questions in both fields, including whether the human RSC processes information about anticipatory head movement and egocentric speed.

## Conflict of interest statement

The authors declare that the research was conducted in the absence of any commercial or financial relationships that could be construed as a potential conflict of interest.
